# Home Features and Assistive Technology for the Home-Bound Elderly in a Thai Suburban Community by Applying the International Classification of Functioning, Disability, and Health

**DOI:** 10.1155/2017/2865960

**Published:** 2017-06-01

**Authors:** Supawadee Putthinoi, Suchitporn Lersilp, Nopasit Chakpitak

**Affiliations:** ^1^Department of Occupational Therapy, Faculty of Associated Medical Sciences, Chiang Mai University, 110 Intawaroroj Rd., Sripoom Subdistrict, Meung, Chiang Mai 50200, Thailand; ^2^Chiang Mai University International College, Chiang Mai University, 239 Huaykaew Rd., Chiang Mai 50200, Thailand

## Abstract

The ageing population is having an impact worldwide and has created a serious challenge in Thailand's healthcare systems, whereby healthcare practitioners play a major role in promoting independent interaction of their client's abilities, as well as environmental factors. The purpose of this study was to survey features of the home and assistive technology (AT) for the home-bound elderly in the community of Chiang Mai, Thailand. Home evaluation included features inside and outside the home, and AT was based on the International Classification of Functioning, Disability, and Health (ICF) concept. Methods included observation and an interview that were used by the researcher for evaluation. The study found that every home had at least one hazardous home feature such as inappropriate width of the door, high door threshold, tall stair steps, no bedside rail, and inappropriate height of the toilet pan. AT was found in houses as general products and technology for personal use in daily living and for personal indoor and outdoor mobility as well as transportation. Therefore, home features and AT can afford the home-bound elderly independent living within the community. Perspective AT according to the ICF concept could provide a common language for ageing in place benefits.

## 1. Introduction

Ageing is not merely the passage of time. It is the manifestation of biological events that occur over a time span. It is important to recognize that the ageing body changes differently in different people. Some systems slow down, while others lose their “fine-tuning.” As a general rule, slight, gradual changes are common, and most of these are not a problem to people who experience them. However, more dramatic changes might indicate serious health problems, and the United Nations Principles for Older Persons has called for action by luring governments into national programs that cover many areas [1]. The strategy is that active and healthy ageing, older people can remain active and independent that it is a good priority for sustainable management of the effects of global ageing.

Thai society is ageing rapidly due to an increasing elderly population, but active ageing level of Thai older persons is not high [2]. The promotion of healthy, active, and productive ageing services is the challenges and opportunities to implement in Thai context. Chiang Mai in the northern of Thailand has been selected as a pilot project to start implementing a primary healthcare strategy [3]. Additionally, this city is the best practice prototype community and the local administrative unit plays a significant role in promoting the quality of life of older people in the local level [4]. Strengthening the capacity of primary healthcare work is deserved for continuous development to achieve sustainable health for all. Thus, promoting and maintaining good health in the home as well as gaining support from the community are challenging. The problem of elderly people falling is associated with environmental factors and has been of significant importance [5] regarding its potentially high incidence, due to age-related physiological changes [6, 7]. Older people usually spend more time at home [8, 9], where their environment is an important factor in independent living [10]. The home should provide good living conditions that enable the elderly to carry out their daily activities independently. “Ageing in place” is a term that means staying at home or in the community and relates to a sense of identity through independence and autonomy [11]. Successful ageing in place should enable the elderly to carry out basic activities associated with daily living safely and independently participate in social roles and receive personal assistance from caregivers as needed.

The environment is perceived to play a significant role in elderly people experiencing falls [12]. The WHO [7] highlighted that falls can result from environmental hazards and Todd et al. [13] promoted a broad environmental definition encompassing the community in which the elderly live, as well as the environmental challenges they face. The physical environment of a house for the elderly has enormous impact on the safety and functional level of older people. Understanding the risk factors in housing is very important for planning and implementing ageing friendly standards.

The risk of falling relates to personal health and the environment. The elderly have a higher risk of accidents and more severe consequences than younger people, and recovery takes longer for older people after a fall. Therefore, prevention of accidents is the best solution for these people. The environment can facilitate health maintenance and management by supporting health promoting behavior and provision of healthcare services. Environmental modifications, healthcare technologies, and assistive technology (AT) can compensate for limitations in functional abilities by reducing the risk of falling and promoting independent living and well-being [14]. Applying the ageing in place concept leads to the reduction of environmental barriers and paves the way for independent functioning in daily activities. Providing the elderly with a community service is classified as a specific characteristic in each of three groups, that is, healthy elderly, home-bound elderly, and bed-bound elderly [15]. This research focused on the home-bound elderly, who are independent or need partial assistance in performing their daily living activities. This group of elderly also has problems in participating in social activities, while mainly living at home. Environmental modifications can enhance the prevention of home-bound elderly being transformed into bed-bound elderly.

The International Classification of Functioning, Disability, and Health (ICF), which originated from the WHO, intends to specify a useful framework for functioning and disability. The term framework of disability, as specified by the ICF, has focused more on the close connection between the limited experience of disabled people, with their environmental design and structure, and the attitude of the general public in providing a common communicative language [16]. Environmental factors are a component of contextual factors in ICF that act as a facilitator or barrier in the successful functioning of a person [17] and influence individual performance [18–20]. The practical manual of the ICF [21] suggests that a structure can be provided for assessing and managing the home environment of home-bound elderly people.

Environmental factors in ICF can have the effect of improving or obstructing the body function of an individual and their ability to execute an activity or participate in society. ICF is able to serve as an organizing framework for AT outcomes. However, Smith et al. [22] reported that use of ICF does not quantify AT interventions, and the outcomes lack specificity. When considering environmental factors, assessment tools for the elderly can be applied to evaluate a physically built environment that facilitates a range of activities in the area of mobility, as well as participation in areas of community life. The aim of this study was to evaluate environmental factors of the community-dwelling elderly living in urbanization area by applying AT classification categories of ICF to enable more specific treatment or intervention.

## 2. Materials and Methods

This study was conducted as pilot study in Chiang Mai, Thailand, between October 2015 and April 2016. This city was selected as the study site because the local administrative units play a significant role to support activities for healthy ageing and arrange home visits for community healthcare undergoing rapid urbanization. Two local communities, Namprae and Sanklang villages, were selected based on the existing structures of primary healthcare program with a significant role of the home visit for the elderly. The study was a cross-sectional survey of people aged 60 years and older. Lists of home-bound elderly people were obtained from the Health Promoting Hospital. Home-bound elderly people were contacted and visited in their homes. All those who agreed to participate in the study were inspected and assessed for home environmental factors and AT. Ethical approval was given by the Ethics Committee of the Faculty of Associated Medical Sciences, Chiang Mai University.

A survey tool was divided into three parts. Part one was the sociodemographic information on age, gender and marital status, condition of health, comorbidities, and physical disability. Part two was a home assessment checklist, using an observation tool with a room assessment technique, in order to evaluate the hazardous features used for elderly and disabled adults living in the community. Finally, part three consisted of an AT checklist completed for the elderly, in order to assess the listing of classification categories from the ICF.

Criteria for judging home features were determined by the 2005 ministerial decree, which specifies the facilities in buildings for the disabled/physically handicapped and elderly [23] as well as the minimum standard of housing and environment for the Thai elderly [24]. The categories of home evaluation are shown in Table 1.

**Figure 1:**
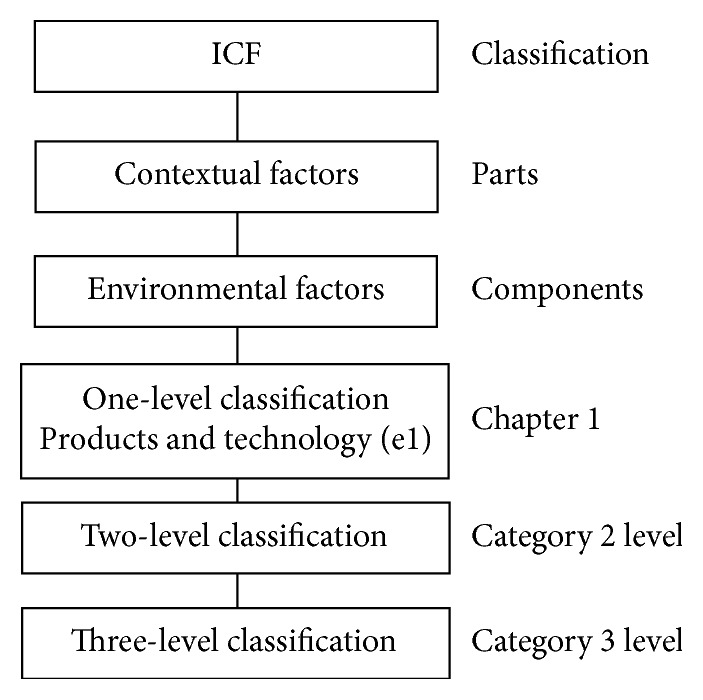
AT checklist based on the ICF framework [16].

The AT checklist was developed as a first version based on the ICF framework under environmental factor components comprising chapter “e1” and a sublevel (category 2 level and category 3 level), as shown in Figure 1. The content validation step was taken by a panel of 3 experts [25], who had at least 5 years qualified experience in teaching and/or practicing in areas of ICF, the community, elderly people, and the environment.

In the first stage to validate the checklist, an expert panel was designed to determine and analyze category variables of the AT of the home-bound elderly. Ten items from all in chapter products and technology were selected. Second stage was carried for rating of agreement by calculating indexes of Item-Objective Congruence (IOC). The value of IOC was higher than 0.5; the item was acceptable. It indicates a good quality for measuring [26]. Finally, six items were validated to be a measurement checklist. The ICF categories are presented in Table 2.

Personal data were collected by face to face interviews with the elderly and/or their family members at home and by direct observation of the home environment. Many techniques were used to complete all of the assessment tools.

## 3. Results

Home-bound elderly people were investigated in the community at Namprae subdistrict. In all, 66 home-bound elderly people (87% of the target population) agreed to participate. Demographic, home evaluation data, and the AT checklist were analyzed by using descriptive statistics to calculate frequency and percentage. The results were divided into three parts: demographic profile of the home-bound elderly, home evaluation, and AT.

### 3.1. Sociodemographic Information

All of the home-bound elderly people had chronic health conditions. The majority of 69.70% of them were female and 57.57% had mobility impairment. Characteristics of the participants are presented in Table 3.

### 3.2. The Home Evaluation

Home evaluation was divided in two parts. The first and second one included assessment outside and inside the home, respectively. The results of home hazard evaluation in the bedroom and bathroom/toilet are shown in Table 4. All of the homes had poor features such as no ramp for a wheelchair and width of the door being smaller than 90 cm. The good features found in the homes were low level floors inside the home and nonslippery floors.

### 3.3. Assistive Technology (AT) in the Houses

The survey of AT in houses of the home-bound elderly is shown in Table 5. AT was found in items e1150, e1201, e125, e1150, and e1151. It did not cover all items of ICF.

AT in homes of the elderly was analyzed in ICF categories as facilitators or barriers as shown in Table 6. Assistive products and technology for personal use in daily living (e1150), and assistive products and technology for personal indoor and outdoor mobility and transportation (e1201), were facilitators for almost all of the participants. The barrier of design, construction, building products, and technology for entering and exiting private buildings (e1550) was found in 87.88% of the homes.

AT device categories listed in ICF categories are presented in Table 7. A majority of 72.73% of the participants had remote controls for a TV, as in the e1150 category. Usability and need were identified as 100% for meeting the need for a walking frame and wheelchair.

## 4. Discussion

In these findings, most of the home-bound elderly people were over 70 years of age and with chronic health conditions and physical disabilities. Perhaps surprisingly, most of their homes had poor features in areas indoors and outdoors, which were barriers in performing activities of daily living. Furthermore, all homes had multiple risks of hazards in rooms and areas where daily routines are performed, such as the bathroom/toilet, kitchen, bedroom, and areas around the house. A strategy for reducing the problems of physical environmental barriers needs to adapt the home environment to enable people with functional limitations to live in their homes as independently as possible. Home modifications such as handrails, stair glides, or grab bars can reduce the chances of elderly people falling [27, 28]. Intervention could (i) make home modifications to eliminate hazards and (ii) construct purpose-built accommodation, especially with AT, to meet the needs of the elderly. However, to determine whether individuals are at risk of falling, the facilities in each individual household must have a falls risk assessment that relates to the elderly person's abilities. There is evidence that environmental hazards are a particularly important fall risk factor among frail elderly people, whose mobility is unstable [29–32].

In this study, the AT in ICF categories on home visits was evaluated. The results showed that e140 (products and technology for culture, recreation, and sport) and e145 (products and technology for the practice of religion and spirituality) were not found in the homes seen. However, AT devices were not covered in all category 3 levels. Although AT plays a role in facilitating independent living for elderly residents in their own homes [33], the main findings of this study revealed a lack of AT devices in many ICF categories. AT has the potential to improve the quality of life for frail elderly people [31, 34]. Surprisingly low number and types of AT were found. Furthermore, all of the AT products were low-tech devices such as a cane, walking frame, or wheelchair. These devices may have shortcomings and limitations and do not cover the daily life activities of home-bound elderly people. This finding is of great importance to the area of environmental intervention. AT needs to develop intervention for home modification and enable the elderly to live in their home independently and for longer periods of time.

This study generated the solution that it is possible to develop AT devices that cover all ICF categories, thus enhancing ageing in place and quality of life. There is research evidence that applying ICF has the potential to identify underlying facilitators and barriers in its human participants [17, 19, 35, 36]. The environmental factors of ICF categories can be used to address and provide a structure of perspective for assessing AT facilities. If healthcare providers in the community use the ICF framework to communicate in the same direction via tools and approaches, it may be possible to help the home-bound elderly to enhance a healthier lifestyle, while overcoming or reducing the barrier of the environment and their physical limitations.

The study was to developing a better measure of the environmental factors of the home-bound elderly people as new approach that can evaluate as either the facilitators or barrier to functioning in the home. Thus, challenge for future research is to use this assessment as a tool at a strategic-level intervention to facilitate ageing in place.

## 5. Conclusions

This study presented a strategy to improve understanding by identifying home hazards using the home evaluation process and evaluating AT in the home by applying ICF. AT is a service or tool that helps the home-bound elderly or disabled to perform their daily activities. Nevertheless, assistive devices can be used to meet the demands of a particular task.

## Figures and Tables

**Table 1:** Characteristics of the home-bound elderly (*N* = 66).

Areas	Items
Outside the home	Area around the house
	Pathway leading to the house
	Exterior/entrances

Inside the home	Condition of the floor
	Movement around the internal area
	Kitchen
	Bathroom/toilet
	Bedroom
	Living/dining room
	Laundry
	Furniture

**Table 2:** Categories of the AT checklist under ICF.

E1 environmental factors	Acceptable consideration related to the home-bound elderly		IOC value
Products or substance for personal consumption (e110)		No	−.40
Products and technology			
For personal use in daily living (e115)	Yes		1
For personal indoor and outdoor mobility and transportation (e120)	Yes		1
For communication (e125)	Yes		1
For education (e130)		No	−.60
For employment (e135)		No	−.60
For culture, recreation, and sport (e140)	Yes		.80
For the practice of religion and spirituality (e145)	Yes		.60
Design, construction, and building products and technology of buildings			
For public use (e150)		No	.00
For private use (e155)	Yes		.60

**Table 3:** Characteristics of the home-bound elderly (*N* = 66).

Characteristics	*N* (%)
Age, years	
60–69	10 (15.16)
70–79	28 (42.42)
>80	28 (42.42)

Gender	
Male	20 (30.30)
Female	46 (69.70)

Marital status	
Single	8 (12.13)
Married	30 (45.45)
Others	28 (42.42)

Chronic health conditions	
No	2 (3.03)
Yes^*∗*^	64 (96.97)

Rated health	
Excellent	—
Good	2 (3.03)
Fair	34 (51.52)
Poor	30 (45.45)

Physical disabilities	
No disabilities	13 (19.70)
Mobility impairment	38 (57.57)
Visual impairment	13 (19.70)
Hearing impairment	2 (3.03)

^*∗*^Chronic health conditions include any of the following; heart disease, high blood pressure, arthritis, asthma, or diabetes.

**Table 4:** Home environment of the home-bound elderly (*N* = 66).

Items	*N* (%)
Outside the home	
Poor features	
No ramp for a wheelchair	66 (100.00)
Uneven/cracked area of ground around the home	10 (15.16)
Good features	
No holes or muddy ground around the home	56 (84.85)
Inside the home	
Poor features	
Width of the door being smaller than 90 cm.	66 (100.00)
High door threshold	64 (96.97)
Tall stair steps	63 (95.45)
No bedside rails	63 (95.45)
Inappropriate height of the toilet pan	61 (92.42)
Door step being higher than 2 cm.	51 (77.27)
Stairs on the staircase being higher than 15 cm.	36 (54.55)
Good features	
Low level floors inside the home	66 (100.00)
Nonslippery floors	62 (93.94)
Adequate lighting	60 (90.91)
No obstacles in the walkway	49 (74.24)
Grab bars in the bathroom	35 (53.03)

**Table 5:** AT in houses of the home-bound elderly.

Environmental factors: products and technology	Having AT in houses of the elderly
For personal use in daily living (e115)	
General products and technology	
For personal use in daily living (e1150)	Yes
Assistive products and technology	
For personal use in daily living (e1151)	No
Products and technology	
For personal use in daily living, other specifications (e1158)	No
Products and technology	
For personal use in daily living, unspecified (e1159)	No
For personal indoor and outdoor mobility and transportation (e120)	
General products and technology	
For personal indoor and outdoor mobility and transportation (e1200)	No
Assistive products and technology	
For personal indoor and outdoor mobility and transportation (e1201)	Yes
Products and technology	
For personal indoor and outdoor mobility and transportation, other specifications (e1208)	No
Products and technology	
For personal indoor and outdoor mobility and transportation, unspecified (e1209)	No
For communication (e125)	
General products and technology	
For communication (1e 250)	Yes
Assistive products and technology	
For communication (e1251)	No
Products and technology	
For communication, other specifications (e1258)	No
Products and technology	
For communication, unspecified (e1259)	No
For culture, recreation, and sport (e140)	
General products and technology	
For culture, recreation, and sport (e1400)	No
Assistive products and technology	
For culture, recreation, and sport (e1401)	No
Products and technology	
For culture, recreation, and sport, other specifications (e1408)	No
Products and technology	
For culture, recreation, and sport, unspecified (e1409)	No
For the practice of religion and spirituality (e145)	
General products and technology	
For the practice of religion or spirituality (e1450)	No
Assistive products and technology	
For the practice of religion or spirituality (e1451)	No
Products and technology	
For the practice of religion or spirituality, other specifications (e1458)	No
Products and technology	
For the practice of religion or spirituality, unspecified (e1459)	No
For private use (e155)	
For entering and exiting private buildings (e1550)	Yes
For gaining access to facilities in private buildings (e1551)	Yes
For ways of finding path routes and designating locations in private buildings (e1552)	No
For private use, other specifications (e1558)	No
For private use, unspecified (e1559)	No

**Table 6:** Facilitators and barriers in ICF coding of homes for the home-bound elderly (*N* = 66).

Items	Facilitator	Neutral	Barrier	NA
Products and technology	*N* (%)	*N* (%)	*N* (%)	*N* (%)
For personal use in daily living				
General products and technology	48 (72.73)	18 (27.27)	—	—
Assistive products and technology	—	—	—	66 (100.00)
Other specifications	—	—	—	66 (100.00)
Unspecified	—	—	—	66 (100.00)
For personal indoor and outdoor mobility and transportation				
General products and technology	—	—	—	66 (100.00)
Assistive products and technology	56 (84.85)	8 (12.12)	—	2 (3.03)
Other specifications	—	—	—	66 (100.00)
Unspecified	—	—	—	66 (100.00)
For communication				
General products and technology	23 (34.85)	—	—	43 (65.15)
Assistive products and technology	—	—	—	66 (100.00)
Other specifications	—	—	—	66 (100.00)
Unspecified	—	—	—	66 (100.00)
For culture, recreation, and sport				
General products and technology	—	—	—	66 (100.00)
Assistive products and technology	—	—	—	66 (100.00)
Other specifications	—	—	—	66 (100.00)
Unspecified	—	—	—	66 (100.00)
For the practice of religion and spirituality				
General products and technology	—	—	—	66 (100.00)
Assistive products and technology	—	—	—	66 (100.00)
Other specifications	—	—	—	66 (100.00)
Unspecified	—	—	—	66 (100.00)
For private use				
For entering and exiting	8 (12.12)	—	58 (87.88)	—
For gaining access to facilities	7 (10.60)	13 (19.70)	—	46 (69.70)
For ways of finding path routes and designating locations	—	—	—	66 (100.00)
For private use, other specifications	—	—	—	66 (100.00)
For private use, unspecified	—	—	—	66 (100.00)

^*∗*^NA: not applicable.

**Table 7:** Assistive technology in the home for the elderly (*N* = 66).

Assistive devices	*N*	Usability				Meeting the requirements	
	(%)	None	Scarce	Average	Frequent	Yes	No
For personal use in daily living							
Dentures	23 (34.85)	7 (10.61)	3 (4.55)	3 (4.55)	10 (15.15)	12 (18.18)	11 (16.67)
Remote controls for a TV	48 (72.73)	18 (27.27)	4 (6.06)	13 (19.70)	13 (19.70)	30 (45.45)	18 (27.27)
For personal indoor and outdoor mobility and transportation							
Cane	33 (50.00)	8 (12.12)	8 (12.12)	8 (12.12)	9 (13.64)	25 (37.88)	8 (12.12)
Walking frame	18 (27.27)	—	—	5 (7.57)	13 (19.70)	18 (100.00)	—
Wheelchair	5 (7.57)	—	—	2 (3.02)	3 (4.55)	5 (7.58)	—
For communication							
Spectacles	23 (34.85)	3 (4.55)	10 (15.15)	5 (7.58)	5 (7.58)	18 (27.27)	5 (7.58)
For entering and exiting private buildings							
Portable ramps	8 (12.12)	—	—	—	8 (12.12)	8 (12.12)	—
For gaining access to facilities in private buildings							
Bedside rails	8 (12.12)	—	—	—	8 (12.12)	8 (12.12)	—
Commode chair	7 (10.61)	—	—	—	7 (10.61)	7 (10.61)	—
